# Supplementary material for: Pyrrolo[3,2-b]pyrrole-1,4-dione (IsoDPP) End Capped with Napthalimide or Phthalimide: Novel Small Molecular Acceptors for Organic Solar Cells
Source: Molecules. 2020 Oct 14;25(20):4700. doi: 10.3390/molecules25204700 (PMC7587392; doi:10.3390/molecules25204700)
Supplement: Supplementary file 1 [file molecules-25-04700-s001.pdf]

Supporting Information

Pyrrolo[3,2-*b*]pyrrole-1,4-dione (IsoDPP) End Capped with Napthalimide or Phthalimide: Novel Small Molecular Acceptors for Organic Solar Cells

Thu Trang Do [1,2], Meera Stephen [2], Khai Leok Chan [3], Sergei Manzhos [4], Paul L. Burn [2,*] and Prashant Sonar [1,5,*]

[1] School of Chemistry and Physics, Queensland University of Technology (QUT), 2 George Street, 4001 Brisbane, Australia; tranghcmut@gmail.com

[2] Centre for Organic Photonics & Electronics, School of Chemistry and Molecular Biosciences, The University of Queensland, 4072 Brisbane, Australia; stephmeera@gmail.com

[3] Institute of Materials Research and Engineering (IMRE), 2 Fusionopolis Way, Singapore 138634, Singapore; khaileok@therightu.com

[4] Centre Énergie Matériaux Télécommunications, Institut National de la Recherche Scientifique, 1650, boulevard Lionel-Boulet, Varennes, QC J3X1S2, Canada; sergei.manzhos@emt.inrs.ca

[5] Centre for Material Science, Queensland University of Technology (QUT), 2 George Street, Brisbane 4001, Australia

* Correspondence: paul.burn@uq.edu.au (P.L.B.); sonar.prashant@qut.edu.au (P.S.)

KEYWORDS: IsoDPP; Napthalimide; Phthalimide; Non-fullerene; Electron Acceptors; Organic Solar Cells

Contents

1. Scheme S1. Synthetic route to DPP isomer

2. Figure S1. (a) ^{1}H NMR (300 MHz, CD_2Cl_2) spectrum and (b) ^{13}C NMR (75 MHz, CD_2Cl_2) spectrum of **compound 5**.

3. Figure S2. (a) ^{1}H NMR (600 MHz, $CDCl_3$) spectrum and (b) ^{13}C NMR (150 MHz, $CDCl_3$) spectrum of **NAI-IsoDPP-NAI**

4. Figure S3. (a) ^{1}H NMR (600 MHz, $CDCl_3$) spectrum and (b) ^{13}C NMR (150 MHz, $CDCl_3$) spectrum of **PI-IsoDPP-PI**

5. Figure S4. AFM images of (a) P3HT**: NAI-IsoDPP-NAI** (1:4) and (b) P3HT**: PI-IsoDPP-PI** (1:1).

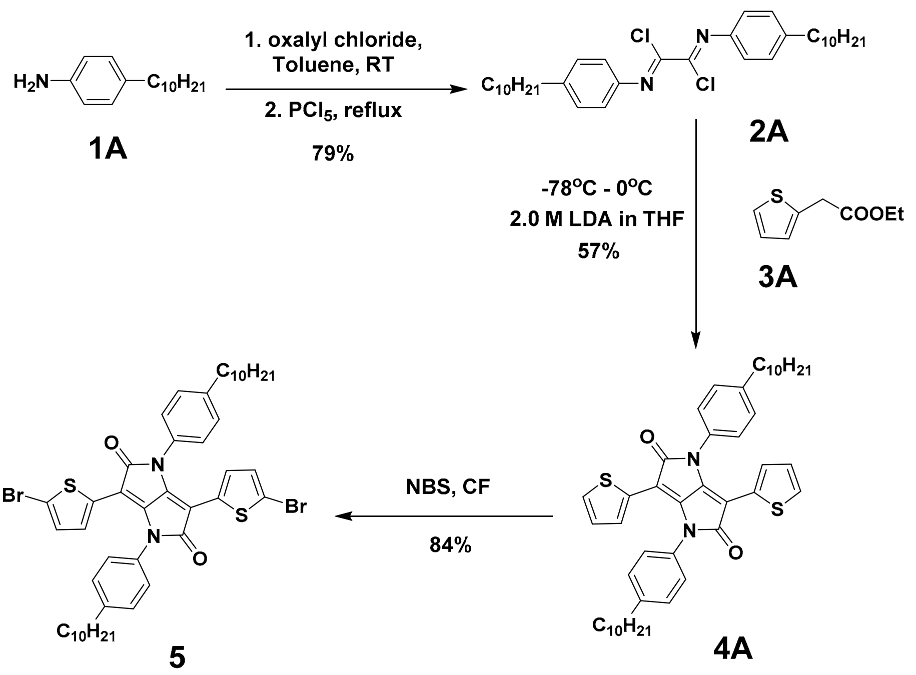

Scheme S1. Synthetic route to DPP isomer

Synthesis of compound 2A: To a solution of 4-decylaniline (**1A**) (5.0 g, 21.4 mmol) in toluene (60 mL) was slowly added oxalyl chloride (1.1 mL, 11.8 mmol) under N_2 at RT. The mixture was stirred for 45 min, then phosphorous pentachloride (PCl_5, 4.5 g, 21.6 mmol) was added and the mixture was heated at 110ºC until no further hydrogen chloride was formed. The reaction mixture was concentrated under reduced pressure and crude product was recrystallized from hexane/CH_2Cl_2 to afford compound **2A** as a yellow crystal (4.74, 79%).

Synthesis of compound 4A: Firstly, ethylthiopheneacetate (**3A**) (2.99 g 17.0 mmol) dissolved in THF at -78ºC and then 2.2 M Lithium diisopropylamide (LDA, 9.36g, 18.7 mml) added dropwise over 30 mins using a dropping funnel. Solution is clear pale orange and it was raised to room temperature for 1 hour then solution turned red after this time. The reaction mixture lowered to -78 °C again and then compound **2A** (4.75g, 8.52 mmol) in 35ml THF added to solution dropwise over 30 mins using a dropping funnel. After addition, cloudy solution raised to room temperature and stirred overnight. Large amount of orange ppt formed in dark brownish/greenish solution. Reaction mixture was filtered, and then washed with ether, ethanol, water, ethanol and ether to give orange cake as compound **4A** (3.57 g, 57 %).

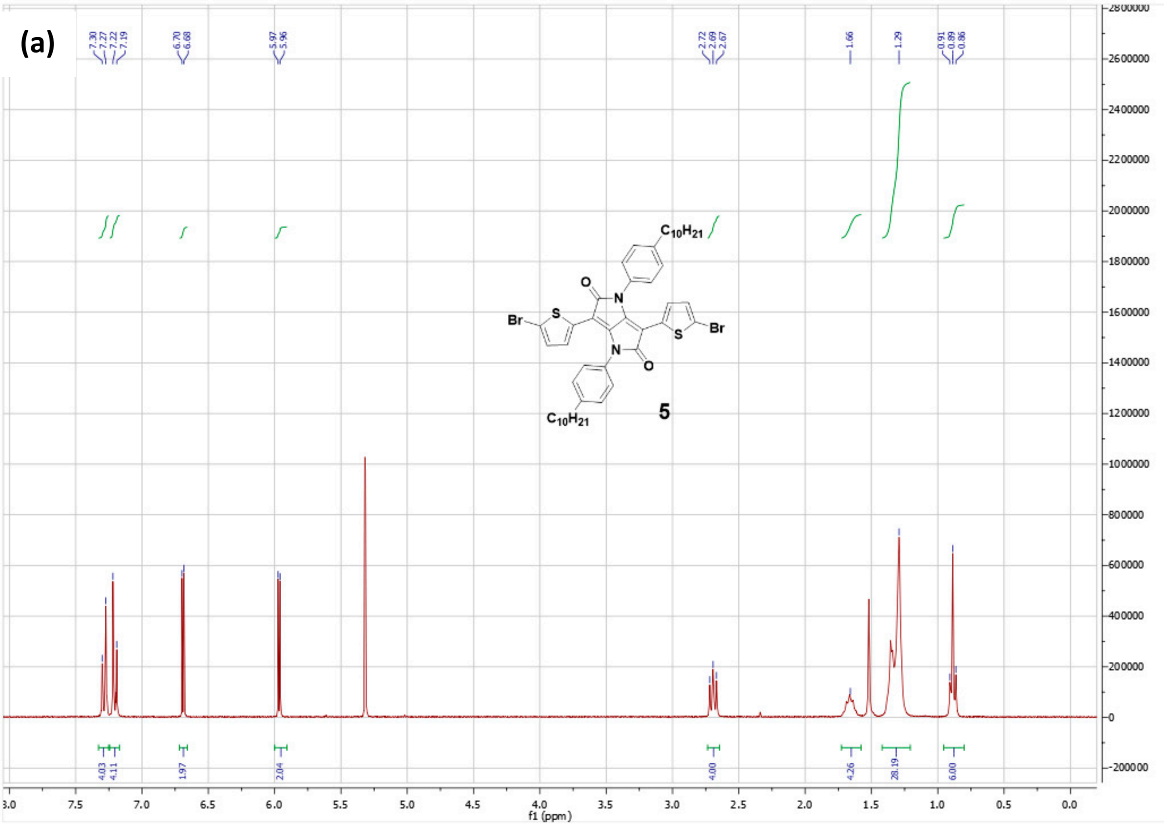

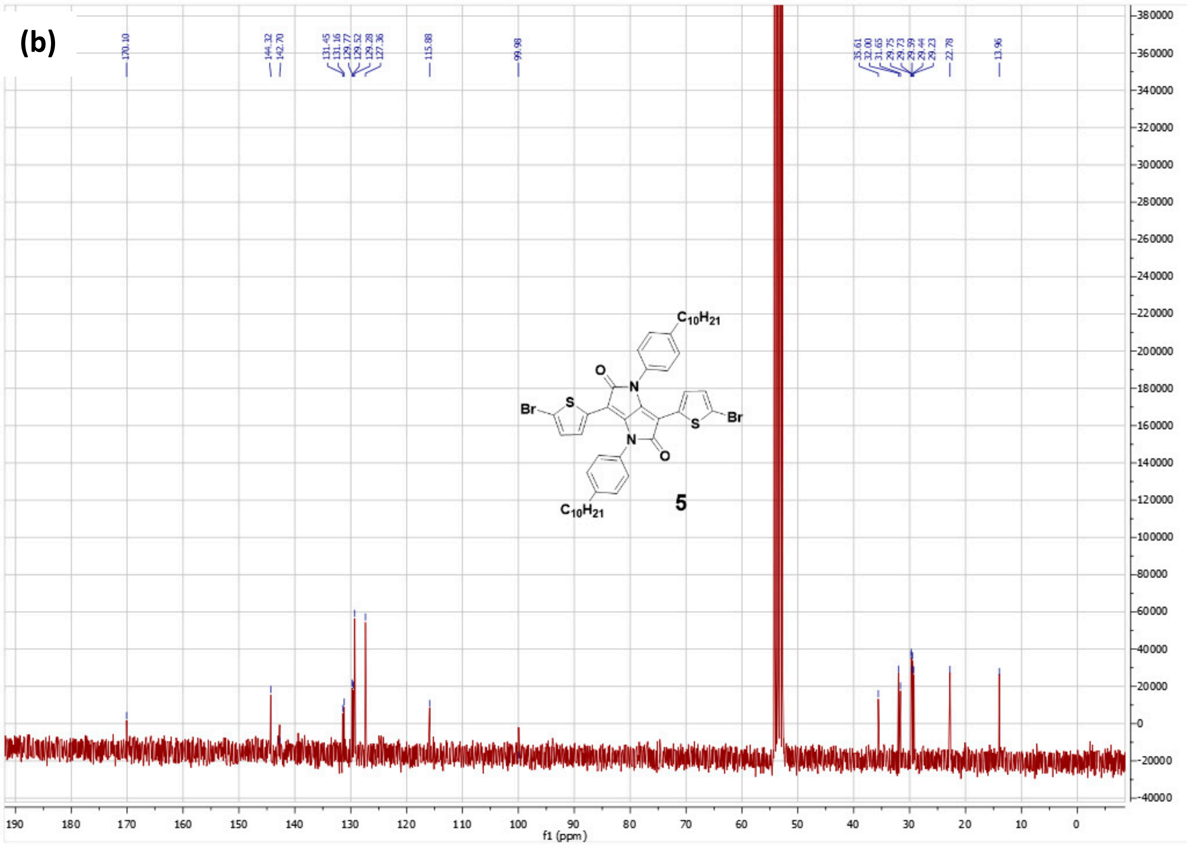

Figure S1. (a) ^{1}H NMR (300 MHz, CD_2Cl_2) spectrum and (b) ^{13}C NMR (75 MHz, CD_2Cl_2) spectrum of **5**

(a)

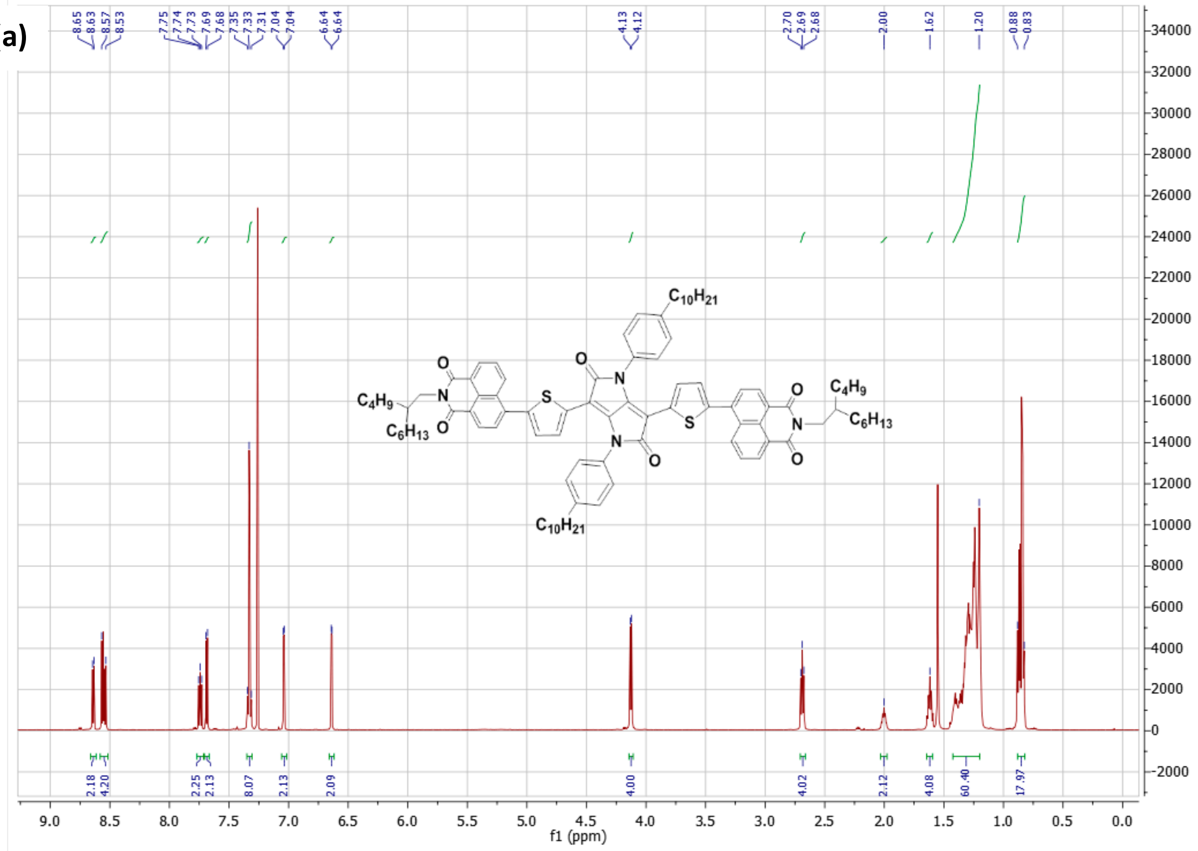

(b)

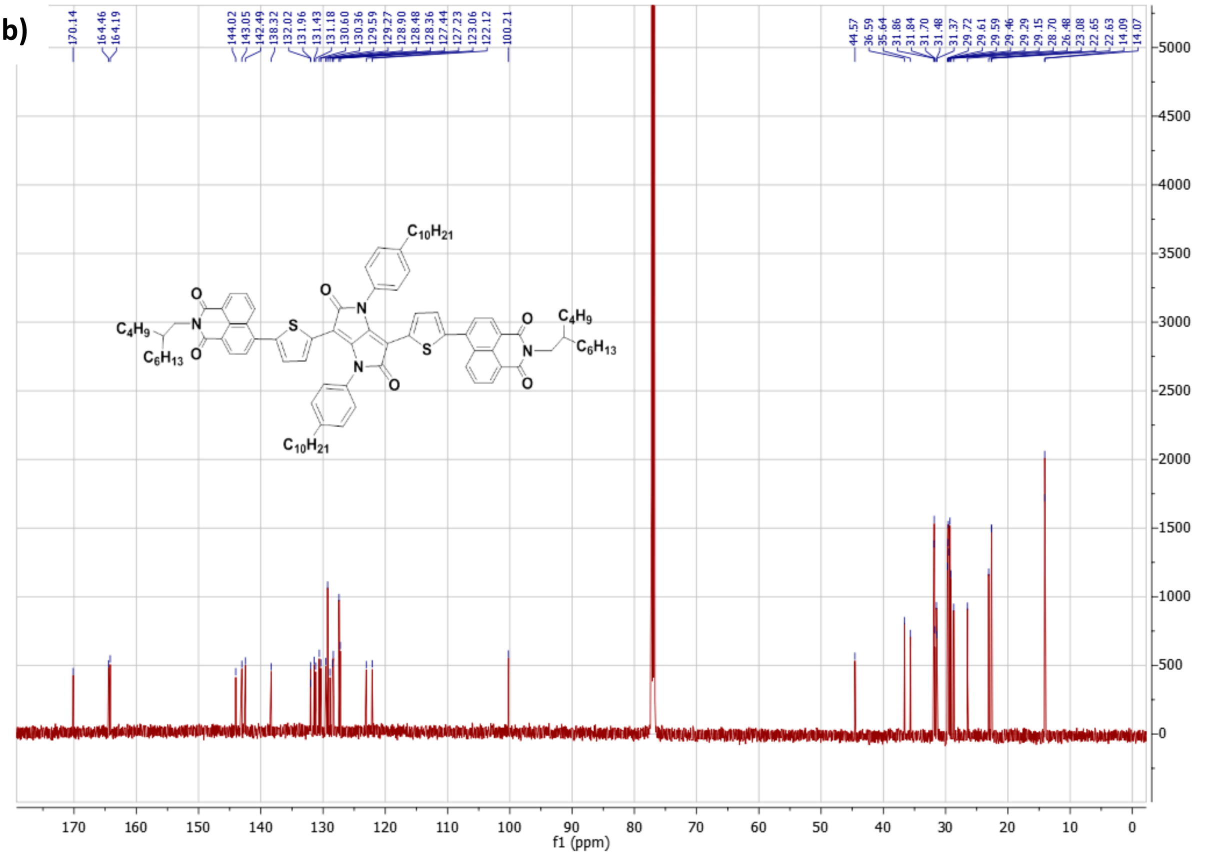

Figure S2. (a) ^{1}H NMR (600 MHz, $CDCl_3$) spectrum and (b) ^{13}C NMR (150 MHz, $CDCl_3$) spectrum of **NAI-IsoDPP-NAI**

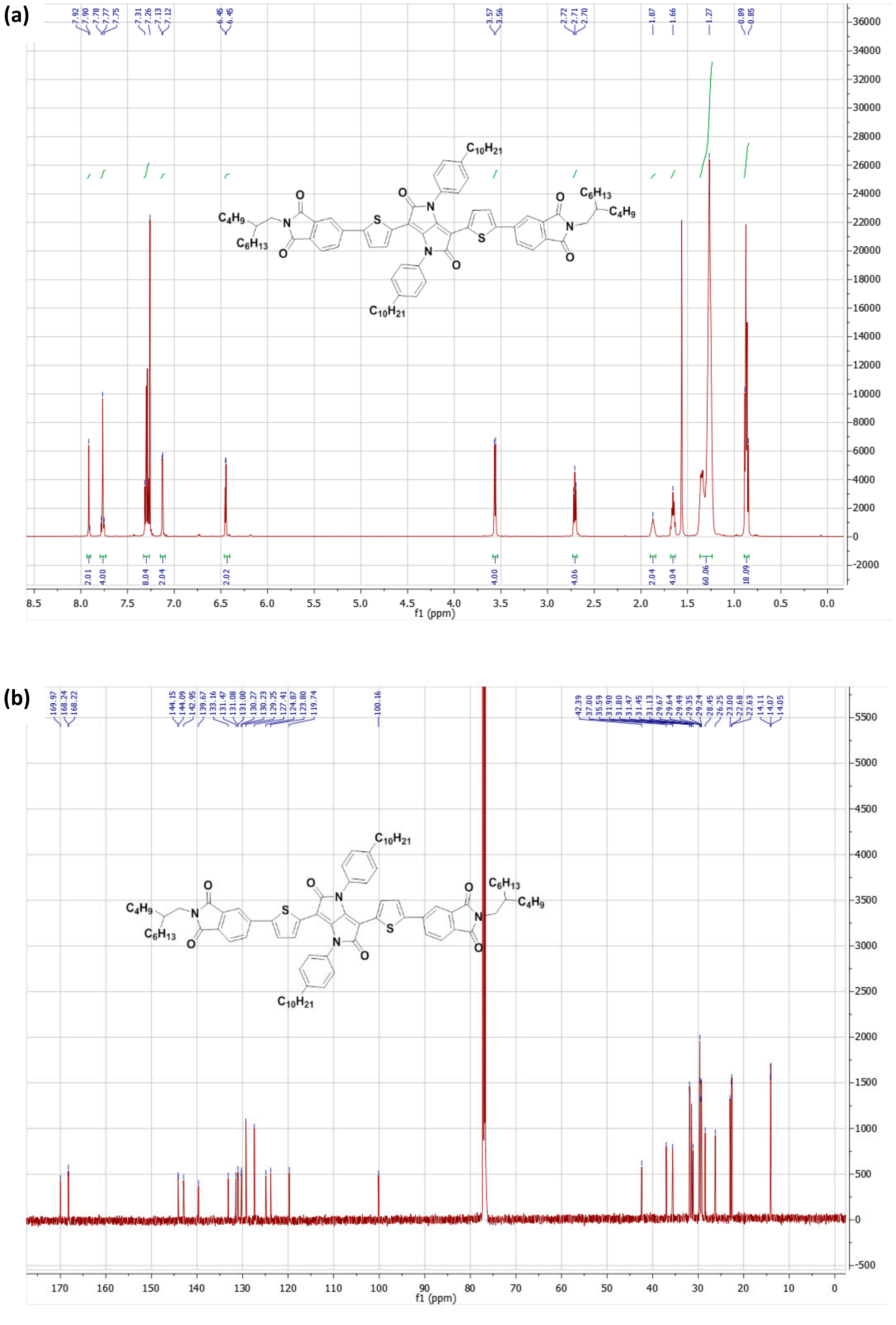

Figure S3. (a) ^{1}H NMR (600 MHz, $CDCl_3$) spectrum and (b) ^{13}C NMR (150 MHz, $CDCl_3$) spectrum of **PI-IsoDPP-PI**

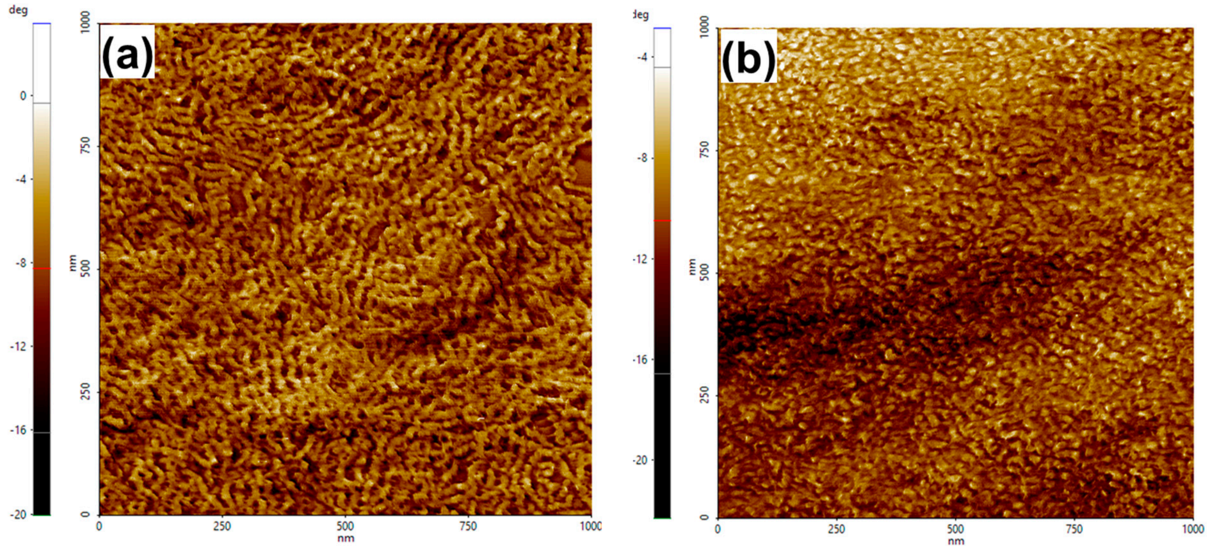

Figure S4. AFM images of (a) P3HT**: NAI-IsoDPP-NAI** (1:4) and (b) P3HT**: PI-IsoDPP-PI** (1:1).
